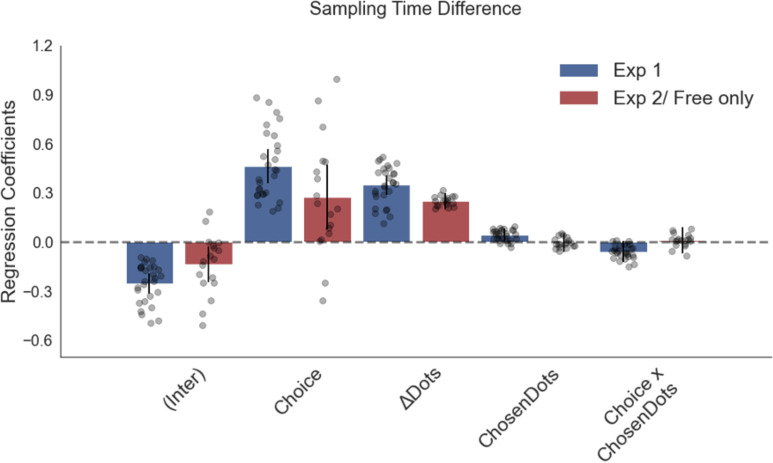# Correction: Humans actively sample evidence to support prior beliefs

**DOI:** 10.7554/eLife.81228

**Published:** 2022-06-28

**Authors:** Paula Kaanders, Pradyumna Sepulveda, Tomas Folke, Pietro Ortoleva, Benedetto De Martino

**Keywords:** Human

Kaanders P, Sepulveda P, Folke T, Ortoleva P, De Martino B. 2022. Humans actively sample evidence to support prior beliefs. *eLife* **11**:e71768. doi: 10.7554/eLife.71768.

Published 11 April 2022

For some of the analyses, z-scores for sampling times of each of the two choice options were computed per participants across both conditions (free and fixed sampling), rather than separately. Of course z-scoring should have been done separately for each of the conditions for all analyses, as the fixed condition imposed sampling times on the participants, while the free condition allowed participants to sample the two choice options freely. These analyses have now been rerun and the affected figures re-plotted using the correct z-scores. This error did not affect any of the main results.

We regret this error and the article has been corrected accordingly.

The corrected Figure 4 is shown here:

**Figure 1:**
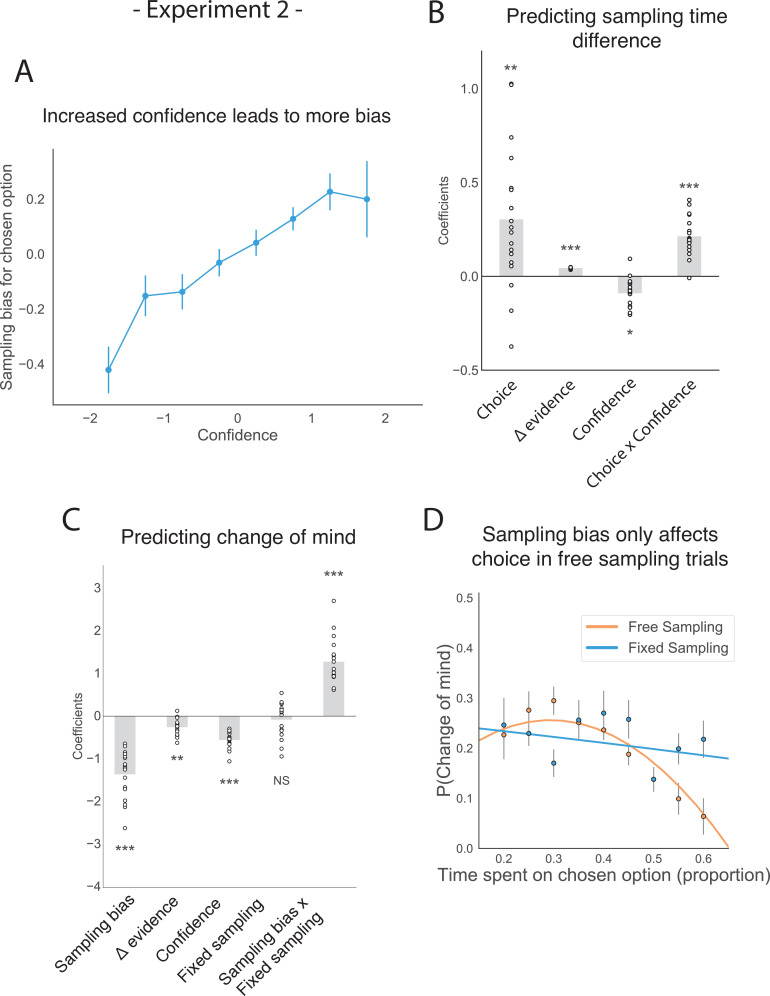


The original Figure 4 is shown here for reference:

**Figure 2:**
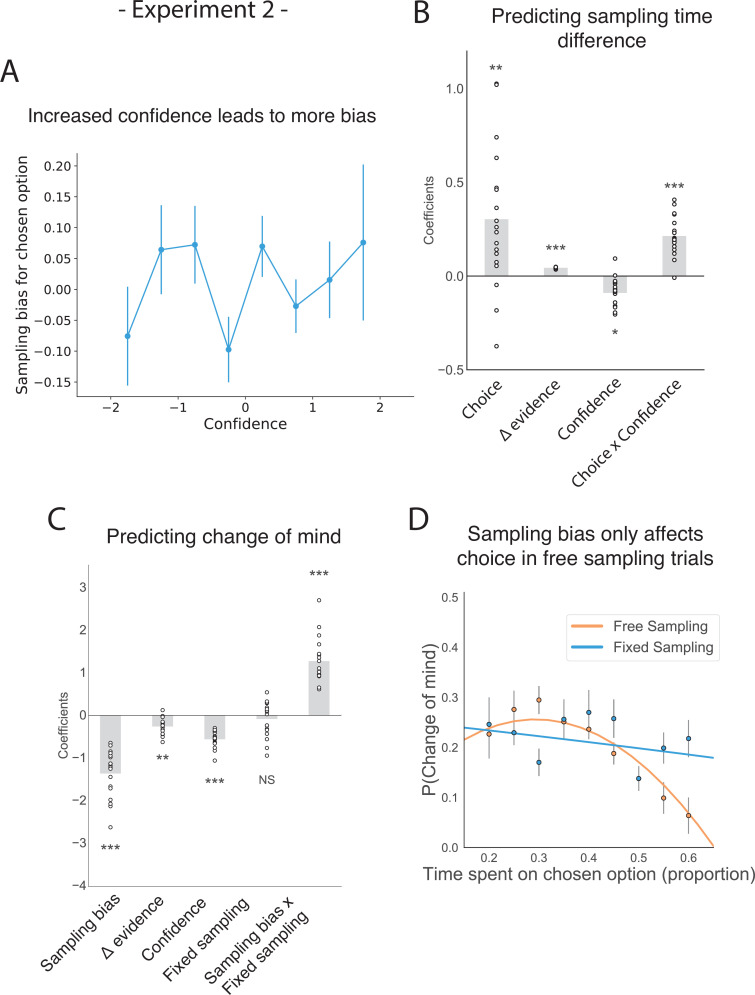


The corrected Figure 2—figure supplement 1 is shown here:

**Figure 3:**
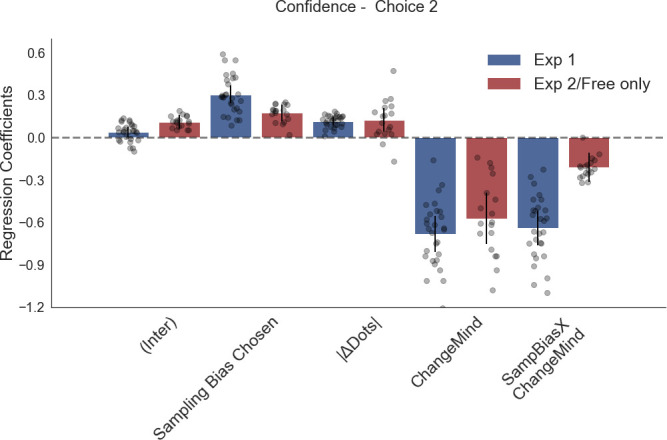


The original Figure 2—figure supplement 1 is shown here for reference:

**Figure 4:**
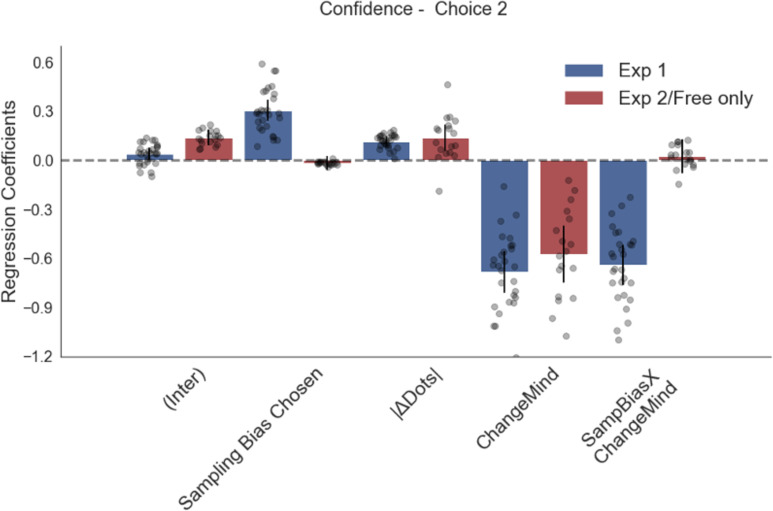


We have also updated a sentence in the Results section about Figure 2—figure supplement 1, from:

“Change of mind also negatively predicted confidence in the second choice phase (Figure 2—figure supplement 1; t_16.59_ = –6.39, *P*<0.001), but in contrast to experiment 1 there was no effect of sampling time difference on the second confidence rating (t_161.30_ = –0.78, *P*=0.44).“

To:

“Change of mind also negatively predicted confidence in the second choice phase (Figure 2—figure supplement 1; t_16.17_ = –6.16, *P*<0.001), and there was again a positive effect of sampling time difference on the second confidence rating (t_17.03_ = 5.79, *P*<0.001) as well as a significant negative interaction effect between change of mind and sampling time difference (t_24.55_ = –3.96, *P*<0.001).”

The corrected Figure 4—figure supplement 3 is shown here:

**Figure 5:**
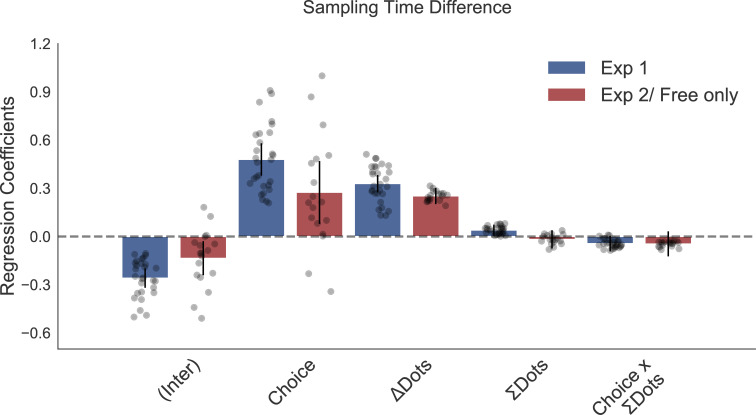


The original Figure 4—figure supplement 3 is shown here for reference:

**Figure 6:**
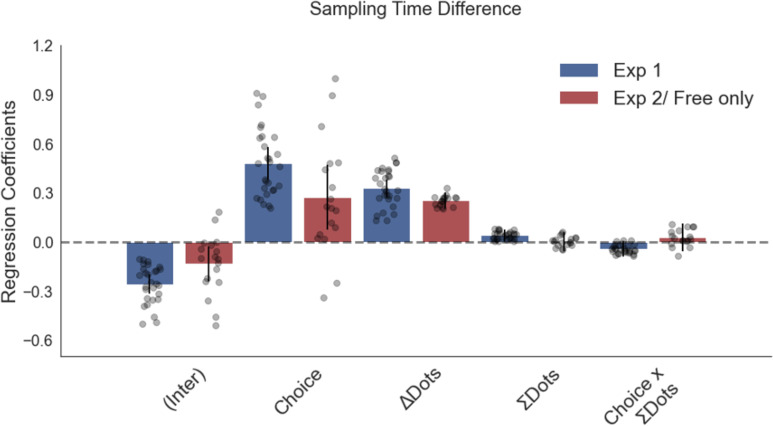


The corrected Figure 4—figure supplement 4 is shown here:

**Figure 7:**
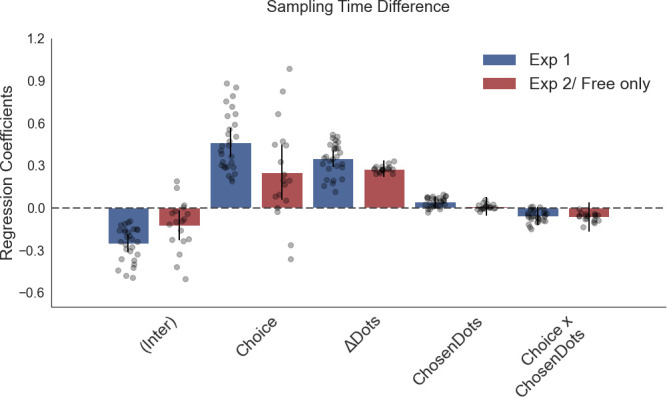


The original Figure 4—figure supplement 4 is shown here for reference:

**Figure 8:**